# Complex bladder and small bowel injury induced by gamma nail fixation: a case report

**DOI:** 10.11604/pamj.2025.51.74.48302

**Published:** 2025-07-11

**Authors:** Ghassane El Omri, Moussaab Rachid, Omar Iraqui Houssaini, Younes Houry, Abdeljalil Heddat

**Affiliations:** 1 Department of Urology, Cheikh Khalifa International University Hospital, Mohammed VI University of Sciences and Health, Casablanca, Morocco

**Keywords:** Trochanteric fracture, gamma nail, bladder injury, bowel perforation, case report

## Abstract

Trochanteric fractures in elderly patients are frequently treated with gamma intramedullary nails. Although commonly performed, this procedure can result in rare but severe complications such as visceral perforation. The case involves an elderly woman who suffered iatrogenic perforation of both the urinary bladder and small intestine during gamma nail insertion. The complication was not immediately recognized, and the patient presented several weeks later with severe sepsis and pelvic peritonitis. Imaging and cystoscopy confirmed the diagnosis. Emergency surgery allowed for small bowel resection and bladder repair, with the nail left in place. The postoperative course was complicated by a vesicocutaneous fistula requiring urinary diversion. This case exemplifies the consequences of insufficient intraoperative imaging during gamma nail fixation in elderly patients. Prompt recognition and multidisciplinary management are essential to reduce morbidity and improve outcomes in such rare but life-threatening scenarios.

## Introduction

Trochanteric fractures are among the most common injuries in elderly patients and are typically managed with gamma intramedullary nailing, a technique favored for its biomechanical stability and minimally invasive nature [1]. Despite its widespread use, the procedure is not without risk. Complications such as malunion, implant failure, and infection are well documented [2]. However, visceral injuries, including perforations of pelvic organs like the urinary bladder or small intestine, are exceedingly rare and often go unrecognized in the immediate postoperative period [3]. These injuries are typically linked to misplacement of the cephalomedullary screw or errors during femoral reaming [4]. The limited number of reported cases in the literature highlights both the rarity and seriousness of such events [5]. Delayed diagnosis may result in severe septic complications, especially in frail elderly patients. We report a case of delayed dual perforation of the bladder and small intestine following gamma nail insertion in an elderly woman.

## Patient and observation

**Patient information**: we report the case of a 78-year-old female with a past medical history of insulin-dependent diabetes mellitus (poorly controlled), hypertension (well controlled), and bilateral knee osteoarthritis. She was functionally independent before the traumatic event and had no history of previous abdominal or pelvic surgery.

**Clinical findings:** upon admission, the patient had a temperature of 39.4°C and showed signs of septic shock: hypotension (blood pressure 76/56mmHg), tachycardia (heart rate 128 bpm), cold extremities, and generalized mottling. Abdominal examination revealed suprapubic tenderness and a palpable pelvic mass.

**Figure 1:**
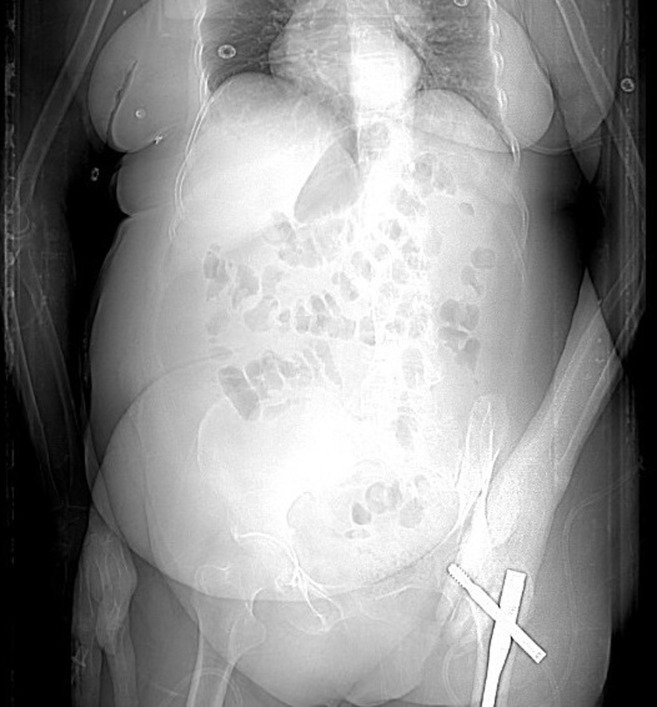
postoperative radiograph showing internal fixation of a pertrochanteric femoral fracture

**Timeline of current episode:** the patient sustained a left pertrochanteric femoral fracture after a fall from standing height. Surgical fixation was performed using a gamma intramedullary nail with a trochanteric plate (Figure 1). The immediate postoperative period was uneventful, and the patient was discharged to a rehabilitation facility. Three weeks later, she presented to the emergency department with pelvic pain and fever.

**Figure 2:**
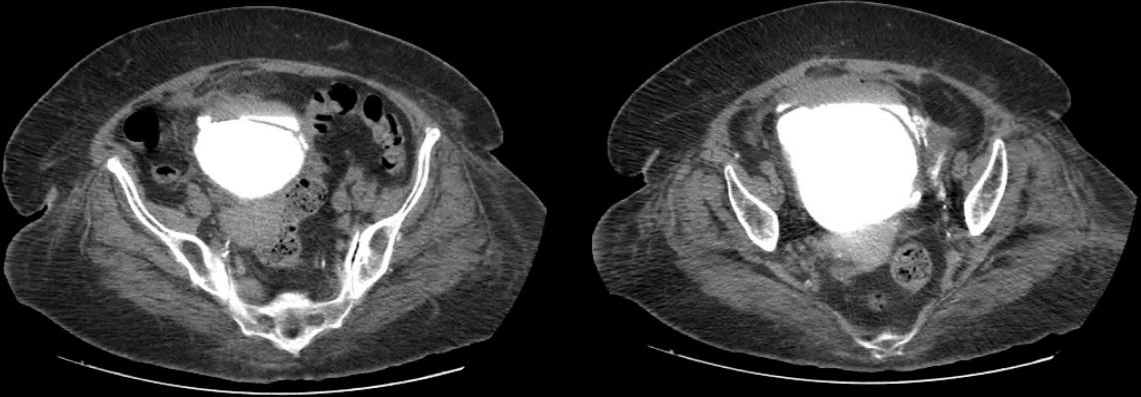
contrast-enhanced imaging showing bladder dome perforation

**Diagnostic assessment:** initial resuscitation was followed by abdominopelvic CT imaging, which revealed pelvic peritonitis and suggested a possible bladder injury (Figure 2). Diagnostic cystoscopy confirmed a complex contused laceration involving the dome and left lateral wall of the bladder, with bile droplets visible in the bladder. These findings were consistent with delayed iatrogenic perforation of both the bladder and the terminal ileum, likely due to hardware misplacement during the initial orthopedic procedure.

**Diagnosis:** the final diagnosis was delayed dual iatrogenic perforation of the urinary bladder and terminal ileum following gamma nail insertion for a pertrochanteric fracture.

**Figure 3:**
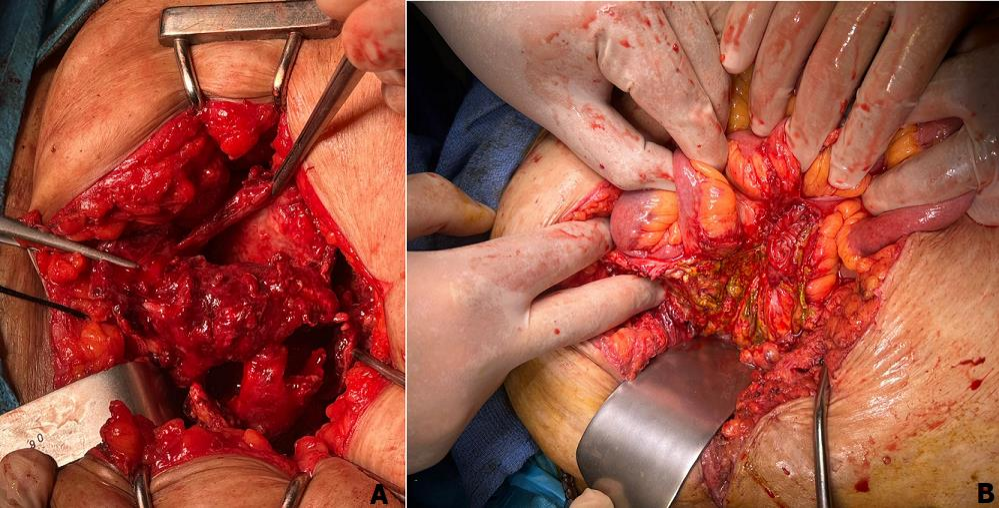
intraoperative findings confirming bladder and ileal perforation: A) multiple lacerations of the bladder involving the dome and left lateral wall with devitalized tissue; B) full-thickness perforation of the terminal ileum with surrounding inflammation

**Therapeutic interventions:** an emergency exploratory midline laparotomy was performed. Intraoperative findings confirmed perforations of the terminal ileum and the bladder (Figure 3). A segmental small bowel resection with stapled end-to-end anastomosis was completed. The bladder was repaired in two layers after debridement of necrotic tissue. A large-caliber urethral catheter was inserted for continuous bladder drainage. Given the stable fracture and surgical difficulty of hardware removal, the orthopedic material was left in situ. Empirical broad-spectrum antibiotics were started and later adjusted based on culture results.

**Figure 4:**
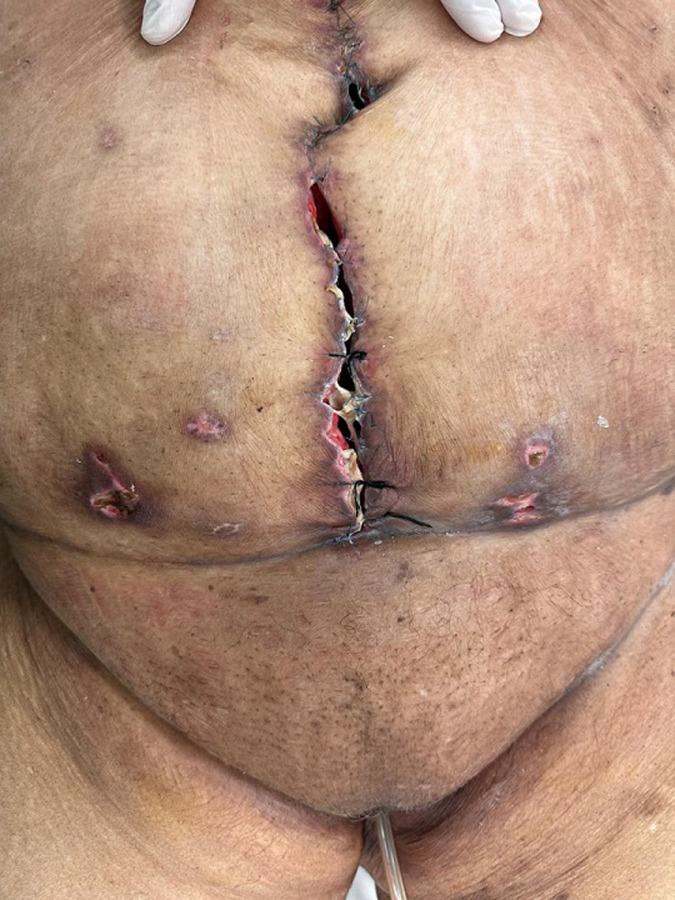
complex vesicocutaneous fistula identified during postoperative assessment

**Follow-up and outcome of interventions:** postoperatively, the patient developed a complex vesicocutaneous fistula (Figure 4). Bilateral cutaneous ureterostomies were performed for urinary diversion. Despite intensive care management, the patient's condition deteriorated due to progressive multiorgan failure, and she died after a prolonged stay in the intensive care unit.

**Patient perspective:** the patient was informed of the complications and prognosis, and remained cooperative throughout her care. Her family was fully briefed on the evolving condition and its associated risks. Despite the unfavorable outcome, the family expressed understanding and acknowledged the quality and diligence of care provided.

**Informed consent:** written informed consent was obtained from the patient's next of kin for publication of this case report and accompanying images.

## Discussion

Trochanteric fractures are common in elderly patients and are frequently managed with intramedullary fixation, particularly using the gamma nail system, which provides stable fixation with minimal soft tissue disruption [1]. Although widely accepted as the gold standard, this technique is not without risks, especially in frail patients with multiple comorbidities. Among the known complications, such as malalignment, hardware failure, and infection, visceral injuries are sporadic but can lead to catastrophic outcomes if unrecognized [2,3]. The literature includes only a handful of reports describing concomitant bladder and bowel injuries during gamma nail fixation, suggesting either a genuinely low incidence or potential underreporting [5].

Visceral injuries during gamma nail placement most commonly involve the urinary bladder, rectum, or small bowel, particularly due to their anatomical proximity to the surgical field. In a systematic review, Anastasopoulos *et al*. described 84 intra-abdominal and pelvic complications during orthopedic procedures, with the urinary tract being the most frequently affected system (33.3%) [6]. These injuries may occur intraoperatively due to technical errors during proximal femoral reaming or incorrect placement of the cervico-diaphyseal screw. Contributing factors include osteoporosis, altered pelvic anatomy, screw misdirection, hardware migration, or poor intraoperative imaging guidance [4,6].

Accurate intraoperative imaging and adherence to technical guidelines are essential to reduce these risks. The screw tip should ideally remain within 5mm of the apex of the femoral head on both AP and lateral views. Freitas *et al*. proposed a screw trajectory assessment model based on cadaveric simulations, showing that medial screw breaches of more than 5mm could place the sigmoid colon or obturator nerve at risk [7]. Improper use of fluoroscopy, failure to assess trajectory, or overemphasis on alignment over depth control can contribute to organ injury. In our case, the postoperative radiographs were considered satisfactory, suggesting that the injury likely occurred during reaming or drilling and went undetected due to subtle early symptoms.

Delayed diagnosis is unfortunately frequent in such cases, as early signs such as pelvic discomfort, fever, or hematuria can mimic routine postoperative events [8]. In this patient, the clinical picture of sepsis three weeks after surgery prompted imaging and cystoscopy, which revealed both bladder injury and signs of bowel involvement, with bile-stained urine strongly supporting ileal perforation. Cystoscopy was particularly helpful in assessing the extent of the bladder lesion. These findings highlight the need for a high index of suspicion in elderly patients with unexplained deterioration after hip surgery.

Management of combined bladder and intestinal injury requires a prompt, multidisciplinary approach. Emergency laparotomy remains the cornerstone to control contamination, resect necrotic bowel, and achieve adequate bladder repair, typically in two layers with absorbable sutures [6]. In this case, the orthopedic implant was left in place due to stable fixation and the absence of signs of infection. When feasible, this conservative approach avoids the morbidity associated with implant removal [6]. However, in the presence of extensive urinary leakage or infection, urinary diversion such as bilateral cutaneous ureterostomies may be necessary.

## Conclusion

This case illustrates a rare but severe complication of gamma nail fixation in a 78-year-old woman, involving delayed iatrogenic perforation of both the bladder and terminal ileum. It highlights the critical importance of intraoperative vigilance and meticulous technique, particularly the continuous use of fluoroscopy not only during guidewire placement but also throughout reaming and screw insertion to verify trajectory and depth. Clinicians must not overlook the possibility of visceral injury in any elderly patient presenting with unexplained pelvic symptoms or sepsis following such procedures. Early recognition and prompt, coordinated surgical management are essential to improving outcomes in these life-threatening scenarios.
